# Lobomycosis Epidemiology and Management: The Quest for a Cure for the Most Neglected of Neglected Tropical Diseases

**DOI:** 10.3390/jof8050494

**Published:** 2022-05-10

**Authors:** Franciely G. Gonçalves, Patrícia S. Rosa, Andrea de F. F. Belone, Léia B. Carneiro, Vania L. Q. de Barros, Rosineide F. Bispo, Yally A. da S. Sbardelott, Sebastião A. V. M. Neves, Amy Y. Vittor, William J. Woods, Gabriel Z. Laporta

**Affiliations:** 1 Graduate Research and Innovation Program, Centro Universitario FMABC, Santo André 09060-870, SP, Brazil; francielygg@hotmail.com; 2 Research Lab at Centro Universitario UNINORTE, Rio Branco 69915-901, AC, Brazil; 3 State Dermatology Program of Acre, Secretariat of Health in the State of Acre (SESACRE), Rio Branco 69917-650, AC, Brazil; leia.borges@bol.com.br (L.B.C.); vannyla_ice@hotmail.com (V.L.Q.d.B.); rosebispo_8@hotmail.com (R.F.B.); yallysbardlott@hotmail.com (Y.A.d.S.S.); william.ac@brturbo.com.br (W.J.W.); 4 Division of Research, Lauro de Souza Lima Institute, Bauru 17034-971, AP, Brazil; prosa@ilsl.br (P.S.R.); abelone@ilsl.br (A.d.F.F.B.); 5 School of Medicine, Federal University of Acre, Rio Branco 69920-900, AC, Brazil; tiaoviana.sebastiao@gmail.com; 6 Division of Infectious Diseases and Global Medicine, Department of Medicine, College of Medicine, University of Florida, Gainesville, FL 32610, USA; amy.vittor@medicine.ufl.edu

**Keywords:** keloidal blastomycosis, Jorge Lobo’s Disease, *Lacazia*, Lacaziosis, lobomycosis

## Abstract

Lobomycosis is a chronic disease caused by *Lacazia loboi*, which is endemic to the Amazon rainforest, where it affects forest dwellers in Brazil. There is no disease control program and no official therapeutic protocol. This situation contributes to an unknown disease prevalence and unmet needs of people disabled by this disease who seek access to treatment. This review provides an update on the subject with an emphasis on therapeutic advances in humans. All relevant studies that addressed epidemiology, diagnosis, or therapeutics of lobomycosis were considered. Seventy-one articles published between 1931 and 2021 were included for a narrative literature review on the epidemiology and quest for a cure. An effective therapy for lobomycosis has been found following decades of research led by the State Dermatology Program of Acre in the Amazon rainforest, where the largest number of cases occur. This discovery opened new avenues for future studies. The main recommendations here, addressed to the Brazilian Ministry of Health, are for lobomycosis to become a reportable disease to ensure that disease prevalence is measured, and that it be prioritized such that affected individuals may access treatment free-of-charge.

## 1. Introduction

Lobomycosis is caused by the as-of-yet unculturable fungus *Lacazia loboi*, which penetrates the skin following traumatic lesions and reaches down to the subcutaneous tissue, thus causing keloid-like nodular lesions in exposed body areas such as the ears, legs, and arms [1]. It affects humans as well as dolphins. In humans, accidental trauma in association with plants, thorns or insect bites is considered a precursor to skin lesions due to *L. loboi*, though not in all cases. As animal-to-human transmission of *Lacazia* has not been confirmed; its main mode of transmission is hypothesized to be traumatic contact with certain tree trunks of tropical rainforests [2]. While the disease occurs throughout Central and South America, it is mainly found in the Amazon rainforest of Brazil. It was first described in 1931 by the dermatologist Jorge Oliveira Lobo in Recife, Brazil, in a case report of a man who had worked several years in the Amazon and developed keloidal skin lesions in the lumbar and gluteal regions. The first dolphin case was reported from the Atlantic coast of Florida in 1971. Lobomycosis in dolphins has also been reported in Europe in 1983 and a decade later in Brazil [3,4,5,6,7].

Reporting of lobomycosis is not a priority for the Brazilian Ministry of Health, therefore disease prevalence is unknown. However, an increase in new cases has been observed over the years at the State Dermatology Program of Acre (SDPA), Rio Branco County, Acre state, Brazil [1,8]. A large hidden prevalence amongst forest people dwelling in remote areas has been suggested as the probable cause of this rise in cases. There is consensus in the literature that effective drug therapy for the treatment of lobomycosis is lacking, and that surgical resection of keloidal skin lesions is often followed by disease recurrence. In more recent years, however, a scientific breakthrough was achieved by observing the outcomes of a natural experiment. This natural experiment consisted of patients co-infected with leprosy and lobomycosis who were treated with the standard therapy for multibacillary leprosy at the SDPA. Not only did symptoms of leprosy disappear during treatment, but resolution of lesions due to lobomycosis was also observed [9,10]. Here we appraise the literature on the epidemiology, diagnosis and management of lobomycosis in a narrative literature review, with an emphasis on therapies.

## 2. Materials and Methods

A narrative literature review was undertaken using PubMed as the primary search engine for the selection of studies. Due to the lack of experimental studies, a systematic review was not feasible. The following medical subject headings (MeSH) were applied to "All Fields" in PubMed Central: Lobomycosis OR Lacaziosis OR Jorge Lobo’s Disease OR Keloidal Blastomycosis AND Epidemiology; AND Diagnosis; AND Therapeutics; AND France; AND Italy; AND Africa; AND Mexico. The first author (FGG) selected all articles relevant to the three axes of interest in this review: epidemiology, diagnosis, and therapeutics.

To expand the search, a complementary strategy for selection of studies was carried out. Google Scholar was used to search for additional references listed in PhD dissertations by an expert on lobomycosis [11].

## 3. Results and Discussion

### 3.1. Case Reports, New Cases, and the Hidden Prevalence of Lobomycosis

The source of lobomycosis infection is unknown. It was long believed that the disease was restricted to Brazil’s Amazon region because of its hot and humid climate, and that male forest workers were the main host population. However, the disease is not only present in Brazil, but also in forested areas of other countries in South and Central America [12,13,14,15,16,17,18]. Intriguingly, lobomycosis also occurs in dolphins [6]. While it would be reasonable to suppose that sylvatic animals such as the sloth, new world monkeys, or the puma might be susceptible to the disease, this has not been borne out.

One of the major shortcomings in our understanding of lobomycosis epidemiology stems from the lack of systematic collection of disease occurrence statistics (e.g., prevalence, incidence). This is not only due to underreporting, which is common in other neglected tropical diseases, but also because lobomycosis is not a reportable disease in Brazil. This has resulted in a large hidden disease burden, insufficient access to necessary health services, and a rising number of patients presenting with advanced clinical manifestations [13,19].

Our understanding of the epidemiology of lobomycosis thus relies heavily on case reports. The first reported case of lobomycosis was from a resident in the Amazon Basin who worked as a rubber tapper in the 1920s. This case was described by the eponymous dermatologist Jorge Oliveira Lobo in 1931, in Recife County in northeastern Brazil [3]. The second reported case was of a 55-year-old man who was also engaged in forest activities (extraction activities, fishing) in Amazonas state, Brazil [20]. Overall, the majority of cases have been reported from the Amazonian forest regions in Brazil (Acre, Amazonas, and Pará states), Peru, Colombia, Bolivia, Ecuador, Venezuela, Suriname, French Guiana, and Guyana [1]. While lobomycosis cases have been reported in other countries, including the United States, Canada, Mexico, Spain, France, Panama, Costa Rica and South Africa, the exposure was hypothesized to have occurred in the Amazon Basin [1,6,16,18,21,22,23].

A longitudinal investigation of lobomycosis was undertaken amongst the Kaiabi indigenous tribe from the 1950s to the 1980s. This tribe is in the northern Mato Grosso state in the Xingu Indigenous Park, which is a well-studied indigenous territory in Brazil. Two adult male brothers were the initial subjects of investigation in 1953. By 1966, 12 cases had been recorded, followed by 15 cases in 1973, 53 cases in 1982, 56 cases in 1986, and 60 cases in 1994 [2,24,25,26,27,28,29,30].

Over the years, case reports of lobomycosis were accumulating from several countries, with the Amazon Basin as the likely source of infection. A total 418 cases had been reported by 1996, of which 255 (61%) occurred in Brazilians [31]. A pioneering case series carried out by Opromolla et al. reported on 40 cases of lobomycosis in Acre state, Brazil, raising the total number of cases reported in Brazilians to 295 [32]. By 2000, 47 additional cases were reported, bringing the total to 465 cases. Of the 465 cases, 295 (63%) occurred in Brazilians. Out of these, 60 (20%) occurred amongst the Kaiabi indigenous tribe [33].

From the scattered epidemiological records constructed mainly by case reports, it would seem that the disease is widespread throughout the Amazon Basin, with some hotspots occurring within this biome. While this observation may be an artifact due to underreporting, given that most infectious diseases show some level of spatial clustering, it would stand to reason that the risk of acquiring lobomycosis would not be uniform throughout the Amazon. The increasing number of new cases arising in Acre state, Brazil, further supports this hypothesis. By way of comparison, 249 lobomycosis cases were reported in Acre state between 1998 to 2008 by the SDPA, while 23 new cases were reported in 1996–2005 in Pará state by the Dermatology Service of the Federal University of Pará state [1,10]. Both Amazonian states have large workforces engaging in forest activities, including the extraction of Açai fruit and Brazil nuts. These activities depend on the conserved habitats of tropical rainforests, but workers do not have direct contact with trees. Although Açai crops are adjacent to continuous forests, workers stay in the crops’ area only. Brazil nut extraction is based on collecting nuts that have fallen on the ground, so there is no need to climb trees. Rubber extraction, however, requires direct contact with the trees, as the rubber tappers move from one rubber tree to the next to collect the sap. The large number of rubber tappers in Acre state may explain why this state has the largest number of lobomycosis cases.

Of the 490 lobomycosis cases reported worldwide by 2006 [1], the distribution was as follows: 318 cases (65%) in Brazil, 50 (10%) in Colombia, 34 (7%) in Suriname, 23 (5%) in Venezuela, 21 (4%) in Costa Rica, 16 (3%) in French Guiana, 13 (3%) in Panama, 4 (0.5%) in Peru, 3 (0.5%) in Bolivia, 2 (0.5%) in Ecuador and in Guyana, and one (0.25%) in Mexico, Europe, the United States, and Canada [1]. While the Amazon basin was the probable source of infection for nearly all cases, at least two cases, reported from South Africa, may have been acquired outside of the Amazon basin [23]. The first case was of a 65-year-old man with keloidal skin lesions of the feet, arms, and face, with a travel history to Mexico. The second case was of a 20-year-old swimmer and diver who frequently visited Palestine and the United Kingdom [23]. In the former case, it can be interpreted that the actual distribution of *L*. *loboi* may be greater than expected, expanding beyond the Amazon basin towards North America. In the latter case, it could be speculated that *L*. *loboi*-contaminated water may confer risk of infection to swimmers or divers.

While the pathogenesis of lobomycosis is poorly understood, the natural history of the disease in humans is characterized by a long incubation period and a slowly progressive, chronic infection. This is likely the result of the subversion of the local immune response [1]. The lengthy incubation period makes it difficult to pinpoint the exact time and location of exposure. Nonetheless, cases of lobomycosis in ecotourists show that transmission can be caused by an acute event (e.g., trauma involving forest debris) dating back years. For example, a 55-year-old Italian man showed infiltrative nodular lesions on the left tibia in August 2016 [19]. The likely origin of this infection was attributed to an exposure to *Lacazia* fungi several years before during a five-day trek in the forests of the Canaima National Park, Bolivar state, Venezuela [19].

The absolute case numbers of Lobomycosis have risen over the years, adding more information, but also more puzzles with regards to its mode of transmission. For instance, a case reported five years ago had the probable location of transmission noted as unknown [34]. The case was of a 36-year-old male farmer who presented with keloid lesions of the left ear. He lived as a farmer in Brazil’s Minas Gerais state, which is located no less than 300 km from the Amazon Basin. He had received visits from Amazonian people at his farm prior to the onset of lobomycosis, raising the question of whether an infected human could have been the source of transmission. Additionally, six new cases of lobomycosis were reported in the Colombian National Army [35]. The disease was probably acquired while in the service in the eastern Colombian Amazon [35]. The duration of illness was between two and 15 years, which suggests that these soldiers were exposed at different times in the same jungle area [35]. Although this jungle area was identified as an infection site for the case series [35], the unknown is where in the environment *Lacazia* fungi reside over the years. Furthermore, a second case of lobomycosis with Mexico as the probable site of infection was reported recently [36], building on the initial case in a South African who had travelled to Mexico [23]. This second individual was a farmer and beekeeper living in southwestern Mexico presenting with multiple nodular lesions in the right ear [36]. In Greece, a histologically confirmed case was reported in a 64-year-old woman without any travel history to Central or South America, marking the first such case from Europe [37]. On the contrary, lobomycosis cases declined amongst the Kaiabi Indians, dropping to only three new cases in the last 20 years [38].

An important outcome from the follow up of lobomycosis cases amongst the Kaiabi Indians between 1965 and 2019 is that a spontaneous cure has not been observed [38]. This means that every new case becomes a prevalent case over time [39]. This, combined with probable high numbers of undiagnosed cases, leads to a mounting burden of disease. The known prevalence today totals 907 cases of lobomycosis in the world [40]. Out of this (*N* = 907), 496 cases (55%) were reported in Acre state, Brazil [40]. In this state, 207 new cases of lobomycosis were reported between 2009–2021. Of these 207 cases, 19 cases occurred in women (9%), the youngest of which involved a 10-year-old child, while the oldest case was of a 106-year-old, and the majority of the individuals affected lived in forested areas [40]. In addition to the known 907 reported cases globally so far [40], it is expected that a larger number remain undiagnosed, contributing to a “hidden prevalence” of lobomycosis.

### 3.2. Lobomycosis in Dolphins and Zoonotic Potential

It had long been believed that lobomycosis was a human disease of Latin American origin until the disease was reported in bottlenose dolphins (*Tursiops truncates*) off the coast of Florida in 1971 [41,42,43]. Due to human interaction with this geographically widespread dolphin species, lobomycosis cases resulting from dolphin-to-human transmission have been reported [43,44,45]. At least two cases were confirmed as zoonotic transmission of lobomycosis. Both cases involved work-related contact with a sick dolphin in which the human patients presented with lesions on their hands months after contact [43,44,45]. Additionally, the possibility of animal-to-human transmission was further implicated when one of the co-authors here (PSR) acquired the disease upon handling experimental mice inoculated with live yeast-like cells from a lobomycosis patient [46]. However, reports of zoonotic transmission are rare and may be more likely in immunocompromised individuals.

Lobomycosis in the bottlenose dolphin is as widespread as the geographical range of the bottlenose dolphin itself, with a prevalence as high as 16% [47,48,49,50,51,52,53,54,55,56,57,58]. Reports showed lobomycosis in bottlenose dolphins off the coasts of Florida, North Carolina (Atlantic Ocean) and Texas (Gulf of Mexico) in the United States, the coasts of Spain and France (Bay of Biscay), and the Brazilian Atlantic coast of Rio Grande do Sul state (Tramandi River) [47,48,49,50,51,52,53,54,55,56,57,58]. The disease has also been reported in the Guiana dolphin (*Sotalia guianensis*) [44]. Interestingly, while the disease occurs readily among dolphins in regions that are not endemic to humans, there is a complete absence of the disease in freshwater dolphins in human-endemic regions, including the Orinoco River in Venezuela and the Amazon River in Brazil [48,49].

Unfortunately, reports of lobomycosis in dolphins are often based on indirect means, such as photographic evidence of lobomycosis-like disease lesions [50]. More than 20 bottlenose dolphins carrying lobomycosis-like disease (LLD) have been photographed in the Indian River Lagoon, Florida [50,51]. Estimates show a LLD prevalence of 3.9% in Guiana dolphins in the Paranagua River Estuary, Paraná state, Brazil [52]. Another dolphin species, *Tursiops aduncus*, was also reported to LLD in the Indian Ocean [53]. The range of LLD prevalence among bottlenose dolphins was estimated as 13.2–16.1% in waters in Central America [54,55,56]. In southern Belize, the first LLD case in Atlantic spotted dolphin, *Stenella frontalis*, has been recorded recently [57]. Photography showing LLD correlates well with lobomycosis in dolphins, as supported by its 75% sensitivity and 100% specificity in comparison with histologic examination of lesion biopsies [50].

### 3.3. Clinical Presentation and Diagnosis of Lobomycosis

The diagnosis of lobomycosis is challenging, as the lesions are often mistaken for cutaneous leishmaniasis, nontuberculous mycobacterial infections including leprosy, sporotrichosis, or other dermatological mycoses [39]. Distinguishing clinical features of lobomycosis include slowly progressing keloidal nodules, which may ulcerate or develop a verrucous appearance over time. Other presentations include hypo- or hyperpigmented macules and papules. Lesions may be pruritic or cause a burning sensation, and they can be isolated or disseminated, and are usually localized in the lower limbs, followed by the ears, upper limbs, and head [59,60]. In the disseminated form, body deformities, an intense pruritus, and ulcerations are commonly observed [61].

Biopsy for histological analysis is considered the gold standard [1,10,17,19,32,59]. *Lacazia* fungal cells are identified by staining with haematoxylin-eosin and Gomori-Grocott methenamine silver stains [32]. Analysis of haematoxylin-eosin dyed papillary dermis by light microscopy at 100× magnification reveals hyperkeratosis, collagen fibroplasia, vascular neoformation, and diffuse inflammatory infiltrate with lymphocytes, epithelioid cells, giant cells, and hemosiderin-laden histiocytes [39]. Reticular dermis dyed by Gomori-Grocott methenamine silver and analysed at 200–400× magnification shows round thick-double-walls yeasts occurring singly or in interconnected chains [39]. These histopathological features can be used for the diagnosis of lobomycosis [32,62].

Vinyl adhesive tape (also known as the Scotch test) can also be used for diagnosis after observation of ulcerated lesions [63]. This test is based on the transepidermal elimination of *L. loboi*, in which fungus is eliminated through the horny layer of the epidermis [62]. This technique consists of the application of vinyl adhesive tape to the scale-encrusted aspect of the lesion, followed by the application of this tape to a glass side present with potassium hydroxide (KOH) and dimethyl sulfoxide (DMSO), and subsequent examination by light microscopy. While the diagnostic accuracy has not been well studied, Miranda et al. confirmed lobomycosis via this technique in five of five patients, and were able to distinguish it from other tropical neglected mycoses (chromoblastomycosis and paracoccidiodomycosis) [63]. Different from lobomycosis, paracoccidioidomycosis and chromoblastomycosis can be cultured in the laboratory [63].

Molecular testing has also been successfully employed for the diagnosis of lobomycosis [19,60,61]. Amplification and direct sequencing of fungal ribosomal RNA genes yielded the diagnosis of lobomycosis in a European man who had travelled to the Amazon region of Venezuela [19]. Another approach has been to amplify the gp43-like gene [60]. However, as molecular testing may not always be available in endemic regions, clinical and microscopic diagnosis remain the most used approaches to identify cases [60,61].

### 3.4. The Quest for a Cure for Lobomycosis

Over the years, numerous antifungal and antibiotic regimens have been attempted with generally unsatisfactory outcomes, falling short of total remission. One studies used sulfadimethoxine 1000 mg/day for an 80-year-old patient in Venezuela in 1961 [64] or sulfamethoxypyridazine 500 mg/day for two cases of 50-year-olds in French Guyana in 1962 [65]. The former study showed partial remission of skin infiltrations and nodules [64], while the latter showed no clear resolution of skin lesions [65] (Table 1). An experimental approach using ketoconazole 400 mg/day showed a decrease in the number of *Lacazia* fungi and mild to moderate remission of skin lesions [66]. In another study (1980), ketoconazole 200 mg/day for six months given to a 45-year-old farmer in Brazil resulted in an unsatisfactory outcome with no cure [67] (Table 1).

After therapeutic studies undertaken by Opromolla et al. in the 1990s [32,63], clofazimine and itraconazole were considered for lobomycosis treatment. Treatment with clofazimine and itraconazole for one year in a 46-year-old Brazilian male was reported to result in the total remission of skin lesions [68] (Table 1). The success of this treatment regimen may be attributed to this patient’s localized facial lesion [68], which is less complex than treating the disseminated forms of the disease [10,69]. In another case involving a localized skin lesion of the left ear, a 29-year-old male forest ranger in Peru was treated with posaconazole 400 mg twice a day for 27 months [70]. Although it resulted in the remission of skin lesions, *Lacazia* fungi were still viable after treatment [70]. However, even after four years of follow up of this case, the disease had not recurred [70] (Table 1). In another successful case, a patient was initially treated with itraconazole 200 mg/day and cryotherapy for seven months [34]. As lobomycosis re-appeared, he had further surgery along with clofazimine (100 mg/day), itraconazole (200 mg/day) and cryotherapy with liquid nitrogen for two years [34]. At the time of the study’s end, the complete remission of skin lesions and the absence of fungi in a biopsy were seen [34] (Table 1).

**Figure 1:**
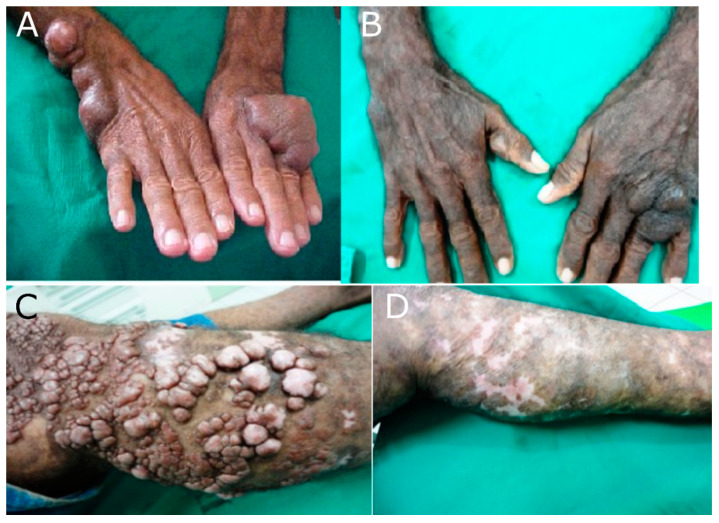
WHO/MDT/MB standard protocol for lobomycosis treatment, SDPA, Acre state, 2020 [71]. (**A**) localized lobomycosis before and (**B**) after WHO/MDT/MB treatment for four years. (**C**) disseminated lobomycosis before and (**D**) after WHO/MDT/MB treatment for four years plus lesion resection twice a week for one year.

Notably, combination therapy with itraconazole clofazimine, rifampin, dapsone, and surgical excision resulted in a clinical cure in both localized and disseminated forms of the disease [46,63,71]. To date, the most promising approach to lobomycosis was found by chance while treating leprosy patients co-infected with lobomycosis in the Leprosy Elimination Program carried out by SDPA in Acre state, Brazil. Ten co-infected patients were treated with the standard protocol for multibacillary (MB) leprosy with multiple drug therapy (MDT; rifampin, clofazimine, and dapsone) as recommend by the World Health Organization (WHO) [10]. Patients reported reduced itching and softening of the skin lesions. Patients were periodically evaluated, and followed up with biopsy and fungal viability assessments [10]. All lesions showing atrophy were then surgically excised, with no further disease recurrence, resulting in 10 of 10 patients cured [10]. Recently, SDPA reported a randomized clinical trial with multibacillary multidrug therapy (MDT/MB/WHO) and surgery resulting in a likelihood ratio of 2.5 (CI 95% 1.4–4.4) of cure compared with untreated or incompletely treated patients (controls) [71] (Table 1). In this trial, out of 80 patients treated with MDT/MB/WHO, 72 (90%) showed improvement, and 20 (25%) were considered cured [71]. MDT/MB/WHO alone is effective in most lobomycosis cases (Figure 1A,B). MDT/MB/WHO associated with surgery can even lead to cure in the disease’s disseminated forms (Figure 1C,D).

Lastly, a 57-year-old Brazilian man showing the disseminated form of lobomycosis was treated with posaconazole (400 mg/twice daily) for 30 months [72]. This patient had previously undergone treatment with itraconazole, dapsone, and clofazimine with no remarkable success. A regimen of posaconazole decreased skin lesions in size and healed some of them, with no side effects [72] (Table 1). This further shows that posaconazole is a potential adjuvant drug for lobomycosis therapeutics.

**Table 1:** Summary of studies on the treatment of lobomycosis, 1960–2021.

Drug	Dosing (Daily) *	Duration ^@^	*n* Patient	Follow-Up Time ^%^	Outcome ^#^	Surgery Used	Side Effects ^$^	% of cure	Ref
Sulfadimethoxine	0.5–2 g	11 d	1	Not done	2	No	No	-	[64]
Sulfadimethoxine	0.25–0.5 g	18 d	2	Not done	1	No	No	-	[65]
Ketoconazole	0.2–0.4 g	90 d	1	Not done	2B	No	No	-	[66]
Ketoconazole	0.2 g	180 d	1	Not done	1	No	No	-	[67]
Clofazimine Itraconazole	0.1 g 0.1 g	1 y	1	2 years	3C	No	Yes^1^	-	[68]
Clofazimine Dapsone Itraconazole	0.05 g 0.1 g 0.2 g	1 y	1	Not available	3C	Yes	No	-	[46]
Posaconazole	0.8 g	27 m	1	4 years	3B	No	Yes ^2^	-	[70]
Itraconazole Clofazimine Cryotherapy with liquid nitrogen	0.2 g 0.1 g every 3 months	2 y	1	Not available	3C	Yes	No	-	[34]
Clofazimine Dapsone Clofazimine Rifampin Dapsone Itraconazole	0.05 g 0.1 g 0.3 g/m 0.6 g/m 0.3 g/m 0.2 g	4 y	103	2 years	3C	Yes	Yes ^1^	25%	[71]
Posaconazole	0.8 g	30 m	1	Not available	2	No	No	-	[72]

*: doses are in grams (g) and daily, except when informed in months (m). ^@^: duration of treatment in days (d), months (m), or years (y). ^%^: Follow-up time after the treatment’s end. ^#^: outcomes of skin lesions: 1 = no resolution, 2 = partial resolution, and 3 = clinical cure. ^#^: outcomes of fungal viability: A = unchanged; B = decreased; and C = clinical cure. ^$^: side effects: Yes ^1^ = skin pigmentation due to the use of clofazimine; Yes ^2^ = headache.

The WHO/MDT/MB therapy (clofazimine, rifampin, dapsone) in Gonçalves et al. [71] was associated with less than $100 in medical expenditure per patient. Thus, this triple drug therapy in combination with surgery not only leads to remission of skin lesions, but also does so at reduced cost (Figure 1). We conclude by advocating for the inclusion of lobomycosis in the Brazilian Ministry of Health in the list of reportable diseases and the WHO program for control of neglected tropical diseases.

## 4. Conclusions

Lobomycosis is the most neglected of the neglected tropical diseases, with a rising number of new cases among residents of the Amazon rainforest. The state of Acre in Brazil has the highest prevalence of lobomycosis in the world, possibly due to the long history of economic development based on rubber tapping. Lobomycosis can cause physical disability, disproportionately affecting low-income heads-of-household who depend on manual labour to provide for their families. This narrative review highlights the role of triple drug therapy with clofazimine, rifampin and dapsone with adjunctive surgical excision as a cost-effective and proven cure for lobomycosis. We recommend that lobomycosis be included in the list of reportable diseases and for the adoption of multibacillary multidrug therapy for the standard treatment of this disease.

## Data Availability

All data used here are contained within the main text.
